# Influence of Plasma Assistance on EB-PVD TBC Coating Thickness Distribution and Morphology

**DOI:** 10.3390/ma18174109

**Published:** 2025-09-01

**Authors:** Grzegorz Maciaszek, Krzysztof Cioch, Andrzej Nowotnik, Damian Nabel

**Affiliations:** 1 Faculty of Mechanical Engineering and Aeronautics, Rzeszów University of Technology, 35-959 Rzeszów, Poland; g.maciaszek@prz.edu.pl (G.M.);; 2 Doctoral School, Rzeszów University of Technology, 35-959 Rzeszów, Poland

**Keywords:** thermal barrier coatings, TBCs, electron beam-physical vapour deposition, EB-PVD, plasma assistance, hollow cathode

## Abstract

In this study, the effects of plasma assistance on the electron beam physical vapour deposition (EB-PVD) process were investigated using an industrial coater (Smart Coater ALD Vacuum Technologies GmbH) equipped with a dual hollow cathode system. This configuration enabled the generation of a plasma environment during the deposition of the ceramic top coat onto a metallic substrate. The objective was to assess how plasma assistance influences the microstructure and thickness distribution of 7% wt. yttria-stabilised zirconia (YSZ) thermal barrier coatings (TBCs). Coatings were deposited with and without plasma assistance to enable a direct comparison. The thickness uniformity and columnar morphology of the 7YSZ top coats were evaluated by scanning electron microscopy (SEM) and X-ray diffraction (XRD). The mechanical properties of the deposited coatings were verified by the scratch test method. The results demonstrate that, in the presence of plasma, columnar grains become more uniformly spaced and exhibit sharper, well-defined boundaries even at reduced substrate temperatures. XRD analysis confirmed that plasma-assisted EB-PVD processes allow for maintaining the desired tetragonal phase of YSZ without inducing secondary phases or unwanted texture changes. These findings indicate that plasma-assisted EB-PVD can achieve desirable coating characteristics (uniform thickness and optimised columnar structure) more efficiently, offering potential advantages for high-temperature applications in aerospace and power-generation industries. Continued development of the EB-PVD process with the assistance of plasma generation could further improve deposition rates and TBC performance, underscoring the promising future of HC-assisted EB-PVD technology.

## 1. Introduction

Thermal barrier coatings (TBCs) have been used in gas turbines since the 1980s to provide heat protection and allow higher turbine inlet temperatures to enhance efficiency. In general, a TBC is a multilayer system on a bulk material consisting of a bond coat, a thermally grown oxide (TGO) layer, and a ceramic top coat [1,2].

Although road maps for “carbon-neutral” engines call for 1900–2000 °C by the early 2030s to unlock further 2–3% cycle efficiency gain and double-digit CO_2_ reductions per flight hour, modern aero and power-generation turbines are already operating at around 1600 °C turbine entry temperature (TET). This drive coincides with a rapidly expanding market for electron beam physical vapour deposition (EB-PVD) TBCs. Global EB-PVD coating revenue is expected to increase from approximately USD 1.5 billion in 2024 to USD 3.2 billion by 2033 [3], a compound annual growth rate of roughly 9%, primarily due to next-generation aircraft engines and hydrogen-ready industrial gas turbines [3].

The effectiveness of thermal barrier coatings produced by the electron beam physical vapour deposition process in aircraft engines is well supported by their unique microstructural characteristics. These coatings are known for their homogeneous columnar structure, which is significant for the strain tolerance of TBCs. The intercolumnar gaps are particularly important for tolerating tensile stresses, erosion, and thermal shock, due to the segmentation of the coatings by these gaps and the compactness of the column microstructure [4].

The microstructural characteristics of these coatings are influenced by several processing conditions, such as substrate temperature, vapour incidence angle, chamber pressure, and deposition rate. For instance, different crystallographic textures can be achieved by varying these parameters, which further affects the coating’s properties [5].

To achieve optimal parameters for the production of these coatings, various techniques have been developed and studied. One promising technology is the EB-PVD process, assisted by plasma. Initially, plasma was used in the production process of the TBC layer during EB-PVD as a key factor in surface cleaning of the substrate before the ceramic layer was applied [6]. Studies show that plasma generated in the EB-PVD process effectively removes contaminants, oxides, and other substances from the substrate surface, leading to improved adhesion of the ceramic TBC layer. When plasma activates the substrate surface, increased adherence of the ceramic layer is achieved, which is of significant importance for the durability of the coating. Another important aspect is controlling the density of the ceramic layer structure using plasma. A review of the literature [7] shows that plasma energy introduces additional movements of material particles, leading to better particle packing and reduction in the layer’s porosity. This process contributes to obtaining a more homogeneous and denser structure, which positively affects the mechanical and thermal parameters of TBC ceramic coatings.

A magnetically enhanced hollow-cathode arc source that injects a low-voltage electron beam (LVEB) of 10–30 eV into the EB PVD plume is commonly used to activate plasma [8]. The LVEB generates plasma densities up to 10^10^–10^11^ cm^−3^ by raising the ionisation fraction of both carrier gas and YSZ vapour to >50 % [9]. Ions that have been accelerated through the substrate sheath arrive with a dose of controlled energy that

- Enhances ad-atom mobility, suppressing sintering gaps and providing a denser, less porous columnar network;
- Improves bond–coat adhesion and in situ surface cleaning;
- Permits bias-mediated steering of column inclination and crystallographic texture (001 vs. 011 fibres);
- Allows for closed-loop rate/stoichiometry control through optical emission feedback, as shown for plasma-activated YSZ evaporation.

When combined, these effects hold promise for combining the compactness and phase stability more commonly associated with PS-PVD coatings with the strain-tolerant architecture of classical EB-PVD.

Zimmermann et al. [10] demonstrated that throttling the argon feed to <20 sccm, while stabilising the discharge with an axial magnetic field, actually raises the external plasma density and extends its reach, because fewer elastic collisions preserve the directed electron beam. Furthermore, spatially resolved Langmuir-probe measurements show that a magnetically stabilised Ta hollow cathode operated at ≈100 A and 0.3 Pa sustains electron densities close to 10^12^ cm^−3^ even 1.25 m downstream of the source, comparable to the length of a modern first-stage turbine blade; this long-range plasma column implies that a single LVEB source can ionise vapour and pre-clean complex air foil geometries without auxiliary plasma guns, simplifying retrofitting into existing EB-PVD chambers.

Although plasma-assisted deposition is a proven principle, its implementation in industrial applications is a challenge. For the following experiments, a dual hollow cathode system designed by the Fraunhofer Institute for Organic Electronics, Electron Beam, and Plasma Technology (FEP) was integrated into an ALD Vacuum Technologies SMART Coater to create an industrial production-like environment and enable a test bench for further improvement. The hollow cathode, a key component in this process, is recognised as a high-performance tool for plasma-activated deposition [11]. It generates an arc discharge plasma containing a high portion of directed electrons with enhanced mean energy, known as the low voltage electron beam (LVEB). This results in the very effective ionisation of gas and vapour particles, leading to high plasma densities, which are essential for high-rate deposition processes. The hollow-cathode plasma-activated deposition (HAD) process is particularly advantageous for the deposition of oxides as abrasion-resistant layers on plastic films and sheets due to its high ion current densities and relatively low compressive stress [12].

Unfortunately, little research has been conducted on the influence of plasma assistance on 7YSZ EB-PVD TBCs. In this paper, 7YSZ EB-PVD TBCs were deposited on Ni-based substrates that are industry standard, both with and without hollow cathode plasma assistance. The thickness distribution and morphology of the 7YSZ top coat were studied. Beyond these observations, the work correlates the key hollow-cathode parameters—discharge current, pressure, and various EB power—with microstructural indicators such as thickness distribution, phase composition, and column inclination angle. By mapping these relationships, a processing window in which plasma assistance not only increases the deposition rate but also produces a denser, more uniform columnar architecture that enhances the thermal-cycle lifetime was identified. The purpose of this paper was to investigate the influence of plasma assistance on 7YSZ TBCs prepared by EB-PVD. These findings are intended to serve as a practical guideline for scaling plasma-assisted EB-PVD from laboratory rigs to full-scale turbine hardware.

## 2. Materials and Methods

To enable a precise characterisation of the thickness distribution of the ceramic top coat, flat plates made of Inconel 718 nickel-based superalloy were selected as the substrate material. The plates had dimensions of 400 mm in length, 160 mm in width, and 5 mm in thickness. Prior to deposition, the substrates were grit-blasted with corundum at a 45° incidence angle from a stand-off distance of 100 mm, followed by ultrasonic cleaning and degreasing in isopropanol. To relieve residual stresses introduced during surface preparation, all plates were subjected to a vacuum heat treatment at 1050 °C for 20 min.

**Figure 1:**
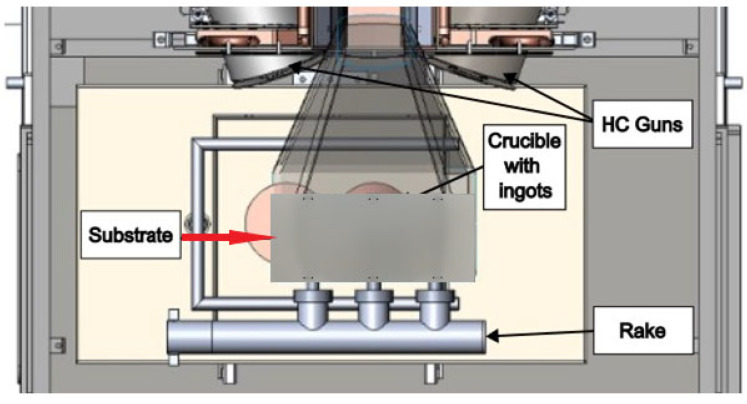
Cross section of coating chamber with integrated hollow cathodes.

**Figure 2:**
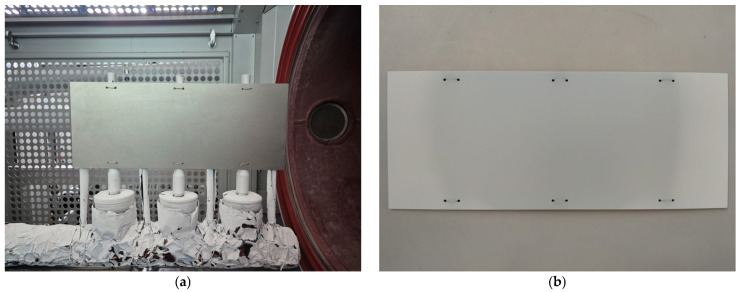
Representative plate (**a**) before the EB-PVD process, (**b**) after the EB-PVD process.

The ceramic top coat, composed of 7 wt.% yttria-stabilised zirconia (7YSZ), was deposited on each plate via the electron beam physical vacuum deposition (EB-PVD) method using the Smart Coater system (ALD Vacuum Technologies GmbH, Hanau, Germany) integrated with a dual hollow cathode plasma assist unit (Figure 1) developed by the Fraunhofer Institute for Organic Electronics, Electron Beam, and Plasma Technology (FEP, Dresden, Germany). The Smart Coater is equipped with a single electron beam gun capable of delivering a maximum power of 160 kW. It allows material evaporation from two crucibles positioned orthogonally to the sample rotation axis and aligned with the primary telescope arm. For the purpose of this study, all substrate plates remained fixed and horizontally aligned above the evaporation source throughout the coating process to ensure controlled and reproducible conditions. A representative plate before and after coating deposition is shown in Figure 2.

The ceramic coating deposition processes were conducted according to the parameters listed in Table 1. In trials 1 and 2, plasma assistance was applied and the electron beam current was adjusted to maintain the substrate temperature throughout the trials. Trial 3 was carried out without plasma assistance at a temperature close to the optimal value for 7YSZ deposition (~950 °C). All other parameters of the EB-PVD process were kept constant throughout all trials. The ingots used in the study were obtained from the same supplier (Phoenix Coating Resources Inc., currently part of Saint-Gobain Coating Solutions, Mulberry, FL, USA) and had diameters ranging from 62.5 to 62.9 mm.

To determine the thickness distribution of the deposited 7YSZ ceramic coatings, a NFe-type gauge (LEPTOSKOP 2050, KARL DEUTSCH, Wuppertal, Germany) was used. Each coated plate was divided into 640 equal squares, each measuring 10 mm × 10 mm. Thickness measurements were taken at the centre of each square, resulting in 640 data points per plate. These measurements were used to generate high-resolution thickness distribution maps. To validate the results, non-destructive measurements from the eddy-current technique were validated with cross-sectional thickness measurements conducted using scanning electron microscopy (SEM, Phenom XL, Thermo Scientific, Waltham, MA, USA). The coating thickness values quoted are an average of 10 measurements.

**Figure 3:**
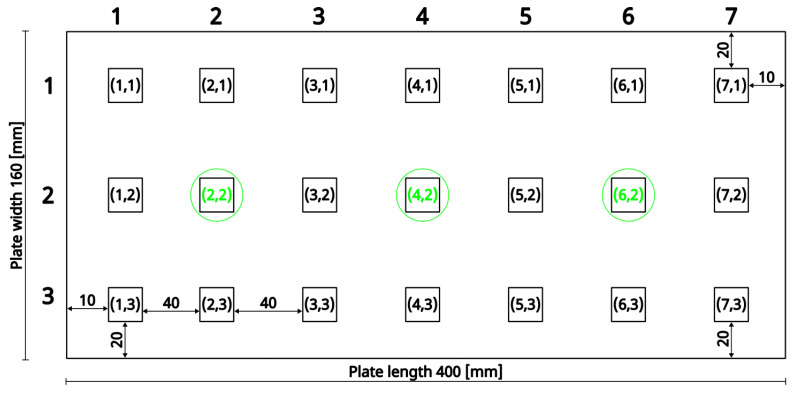
Schematic representation of the coated plate. Squares indicate the regions from which specimens were extracted for further analysis. Green-circled squares indicate the regions from which specimens were extracted for further analysis, including XRD and scratch testing.

To analyse the morphology of the deposited ceramic coatings, small specimens were extracted from selected regions of the plates, as schematically indicated by squares in Figure 3. Surface topography was examined using a scanning electron microscope (SEM, Hitachi S3400, Hitachi High-Tech Co., Ltd., Tokyo, Japan), while phase composition was analysed by X-ray diffraction (XRD). Additionally, metallographic cross-sections were prepared from selected plate fragments and analysed by SEM. Microstructural imaging was performed using a backscattered electron detector (BSE, INCA HKL Nordlys, Oxford Instruments plc, Abingdon, United Kingdom).

To determine the mechanical properties of the deposited coatings, a scratch test was conducted using a Revetest device (CSM Instruments, Peseux, Switzerland). Scratch tests were performed using a Rockwell diamond indenter of 200 µm radius, applying a progressive load from 1 N to 200 N with a loading rate of 39.8 N/min. The measurements were taken over a 10 mm measuring length with a scanning speed of 2 mm/min. All scratch tests were carried out with constant parameters for comparison purposes.

## 3. Results and Discussion

### 3.1. Coating Thickness Distribution Analysis

**Figure 4:**
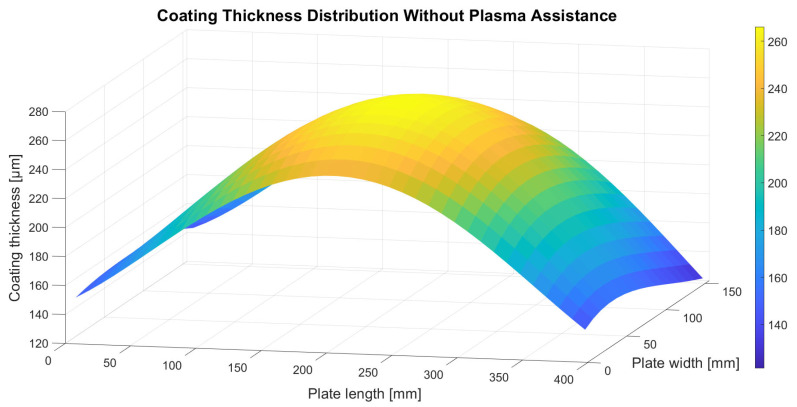
Thickness distribution of the 7YSZ top coat deposited without plasma assistance.

To reduce the impact of local fluctuations and enhance the interpretability of the coating thickness plots obtained using the non-destructive eddy-current method, a two-variable polynomial surface fitting was applied. The original data set consisted of discrete measurements defined as z = f(x, y), collected on a regular grid with dimensions 15 × 40, where x and y correspond to the length and width of the coated plate, respectively, and z represents the measured coating thickness. A fourth-degree polynomial function with respect to both variables was used for data fitting. The thickness distribution of the coating deposited without plasma assistance was approximated using the following polynomial model:

z(x, y) = 0.410503x + 0.166047y + 8.67163·10^−8^x^2^y^2^ + 0.00579135xy − 0.0000514232xy^2^ − 0.00000679474x^2^y + 6.34707·10^−8^xy^3^ − 1.38391·10^−8^x^3^y + 0.00360122x^2^ − 0.0000225088x^3^ + 2.68818·10^−8^x^4^ − 0.00219649y^2^ + 0.0000154303y^3^ − 6.17645·10^−8^y^4^ + 146.983 (1)

where z denotes the thickness of the coating deposited without plasma assistance and x and y correspond to the position coordinates along the length and width of the plate, respectively. The RMSE (Root Mean Square Error) of the generated polynomial fit was 7.96 µm, which indicates good agreement of the model with the measured data. The resulting surface plot of the fitted model is shown in Figure 4.

**Figure 5:**
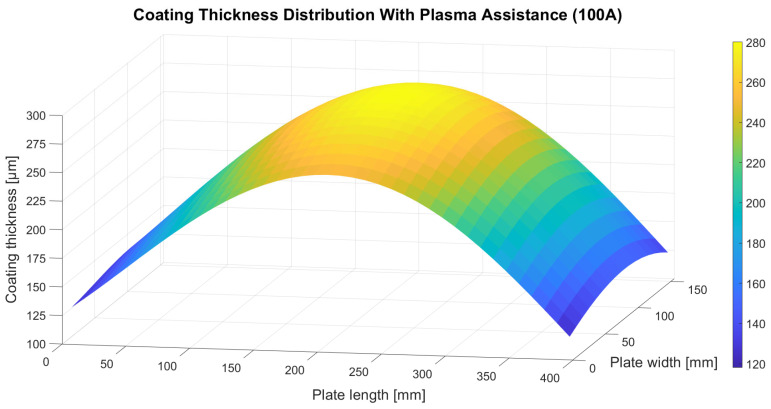
Thickness distribution of the 7YSZ top coat deposited with plasma assistance at 100 A.

The thickness distribution of the coating deposited with plasma assistance using a hollow cathode system operated at 100 A was similarly approximated using a fourth-degree two-variable polynomial model:

z(x, y) = 0.728349x + 0.0556492y + 3.06985·10^−8^x^2^y^2^ + 0.00390898xy − 0.0000207663xy^2^ − 0.00000926967x^2^y + 1.84207·10^−8^xy^3^ + 4.7775·10^−9^x^3^y + 0.00277246x^2^ − 0.0000213282x^3^ + 2.39921·10^−8^x^4^ + 0.0022617y^2^ − 0.0000202651y^3^ + 2.36229·10^−8^y^4^ + 125.973 (2)

where z denotes the coating thickness obtained under plasma assistance at 100 A and x and y represent the coordinates along the length and width of the plate, respectively. The RMSE of the generated polynomial fit was 14.42 µm. The resulting surface plot is presented in Figure 5.

**Figure 6:**
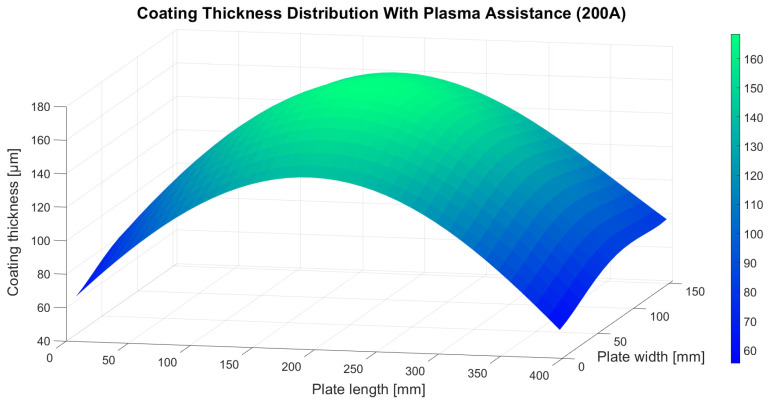
Thickness distribution of the 7YSZ top coat deposited with plasma assistance at 200 A.

The thickness distribution of the coating deposited with hollow cathode plasma assistance at 200 A was also modelled using a fourth-degree two-variable polynomial:

z(x, y) = 0.696912x + 0.154182y + 3.39424·10^−8^x^2^y^2^ + 0.00323704xy − 0.0000112077xy^2^ − 0.0000146163x^2^y − 6.51513·10^−9^xy^3^ + 1.58008·10^−8^x^3^y − 0.000625108x^2^ − 0.00000603415x^3^ + 7.72145·10^−9^x^4^ + 0.00574421y^2^ − 0.0000787418y^3^ + 2.53556·10^−7^y^4^ + 60.8304 (3)

where z denotes the coating thickness obtained under plasma assistance at 200 A and x and y represent the coordinates along the length and width of the plate, respectively. The RMSE of the generated polynomial fit was 5.49 µm. The resulting surface plot is shown in Figure 6.

Analysis of the coating thickness distributions presented in Figure 4, Figure 5 and Figure 6 indicates that plasma assistance does not significantly affect the spatial pattern of ceramic material deposition. In all deposition trials, the maximum coating thickness consistently appeared directly above the evaporation source, located at the geometric centre of the substrate plate. Thickness values systematically decreased toward the plate edges, confirming that the highest flux density of the evaporated ceramic material is concentrated above the crucible and diminishes with increasing lateral distance from the evaporation source. This effect is attributed to the use of a single active crucible in all deposition trials.

In contrast, plasma assistance had a marked influence on the absolute thickness of the deposited coatings. Specifically, the coating produced with hollow cathode plasma operating at a current of 200 A exhibited a significantly lower thickness compared to the coating deposited without plasma support. This reduction can be attributed to the lower electron beam emission current applied in this study (1.62 A) and, more importantly, to the increased hollow cathode plasma current, which generates a substantially higher plasma arc density compared to the other trials. The elevated ion concentration within the plasma effectively forms a barrier layer that hinders the free transport and condensation of the ceramic vapour onto the substrate surface. A comparison between the coating deposited with plasma assistance at 100 A and that deposited without plasma support shows that both coatings exhibit nearly identical average thicknesses. Notably, this similarity was achieved despite differences in electron beam emission current: 2.32 A in the plasma-free process and 1.82 A in the plasma-assisted one. This finding suggests that plasma assistance enables the formation of coatings of comparable thickness at reduced energy input, highlighting the potential economic and operational benefits of the hollow-cathode plasma system implemented in this study.

Table 2 presents a comparison of coating thickness values measured by the non-destructive eddy-current method and cross-sectional observations using scanning electron microscopy (SEM). For coatings deposited without plasma assistance, the overall discrepancy in thickness measurements between the two methods was 3.24%, with the maximum deviation of 7.04% observed at position (6,3). In the case of coatings produced with plasma assistance, the total difference in thickness measurements was 3.71% for the hollow cathode plasma current of 100 A (with maximum deviation of 6.98% at position (3,2)) and 3.13% for the plasma current of 200 A (with maximum deviation of 6.81% at position (3,1)). These results demonstrate a high level of consistency between the two measurement techniques, thereby confirming the reliability of the eddy-current method for quantitative assessment of the coating thickness. Consequently, the measured thickness distributions are considered sufficiently accurate for the purposes of this study.

### 3.2. TBC Coating Morphology Evaluation

X-ray diffraction (XRD) analysis was performed to determine the phase composition of yttria-stabilised zirconia (YSZ) coatings deposited on metallic substrates by electron beam physical vapour deposition (EB-PVD). Measurements were carried out on specimens extracted from predefined positions on coated plates, marked by green circles in the schematic diagram (Figure 3) at coordinates (2,2), (4,2), and (6,2).

**Figure 7:**
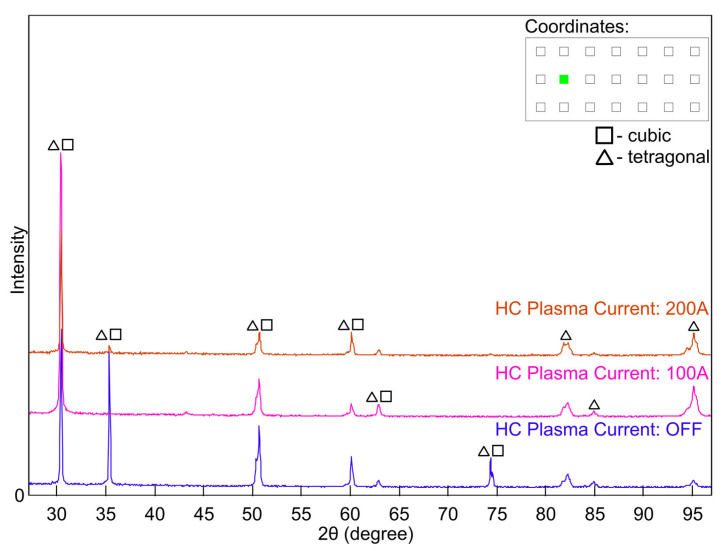
X-ray diffraction pattern (XRD) of the coating sample collected from position (2,2) on the plate.

**Figure 8:**
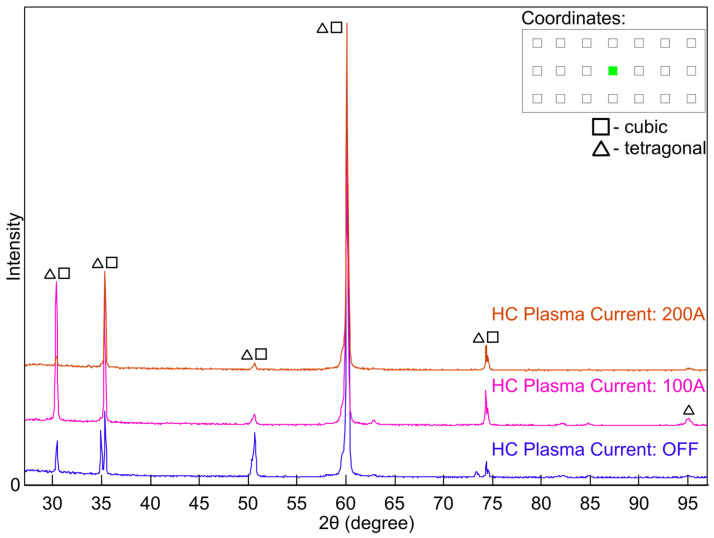
X-ray diffraction pattern (XRD) of the coating sample collected from position (4,2) on the plate.

**Figure 9:**
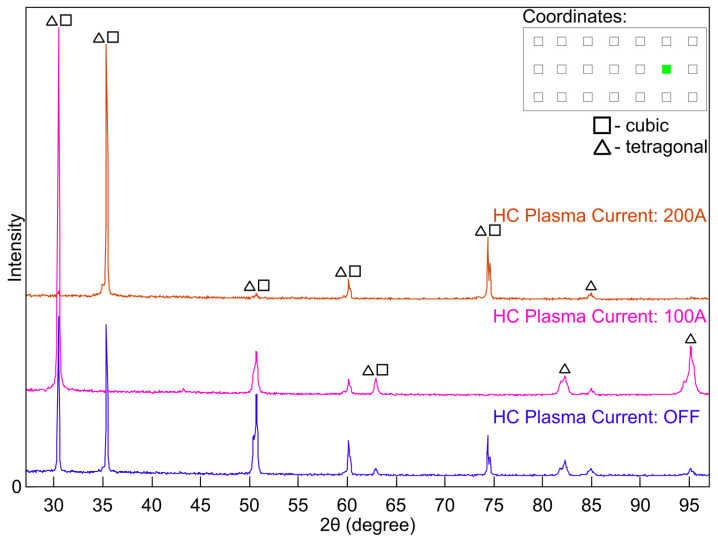
X-ray diffraction pattern (XRD) of the coating sample collected from position (6,2) on the plate.

Diffractograms were collected for coatings deposited under different plasma conditions. For specimens taken from equivalent in-plane locations on the substrates, the corresponding XRD patterns (Figure 7, Figure 8 and Figure 9) show closely matching diffraction peak positions, yet notable variations in peak intensities were observed. The invariance of 2θ peak positions, parameters that are controlled predominantly by the lattice parameter and only marginally affected by typical levels of preferred orientation, confirms that the average d-spacings of the stabilised zirconia lattice remain essentially constant across all processing conditions. In contrast, the pronounced variations in relative peak intensities are symptomatic of plasma-induced modifications to the diffracting volume, resulting from variations in crystallographic texture and accompanying changes in coherent domain size and lattice defect density. Such intensity differences are consistent with changes in crystallographic preferred orientation, diffracting volume fraction, and defect content that can arise from plasma-enhanced adatom mobility and low-energy ion bombardment during growth in the ceramic coatings. The most marked enhancement in the intensity of the diffraction peak was observed in the coating processed with hollow-cathode plasma assistance at 100 A (pink traces in the figures), suggesting improved crystallinity or a stronger texture under these conditions. It should be emphasised that the crystal structure obtained in the plasma-assisted process (100 A) was achieved at 850 °C, that is, notably lower than the 950 °C required in the standard EB-PVD process.

Phase analysis over the 2θ range of 30–95° revealed the predominance of tetragonal and cubic phases of YSZ, in agreement with previously reported behaviour of 7 wt.%Y2O3-stabilised zirconia deposited by EB-PVD [13,14]. Representative diffraction peaks at approximately 30.2°, 34.9°, and 50.2° are characteristic of overlapping reflections from the tetragonal and cubic zirconia phases typical of fully stabilised YSZ coatings [15,16,17]. The SEM micrographs reveal a typical columnar structure characteristic of YSZ coatings produced by EB-PVD. This microstructure is consistent with the presence of a metastable mixture of tetragonal and cubic phases, as indicated by the XRD results.

**Figure 10:**
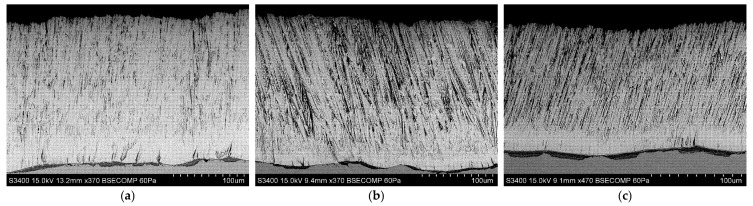
SEM cross-sectional morphology of the 7YSZ top coat deposited on the plate in location (2,2): (**a**) without plasma assistance; (**b**) with plasma assistance (100 A); (**c**) with plasma assistance (200 A).

**Figure 11:**
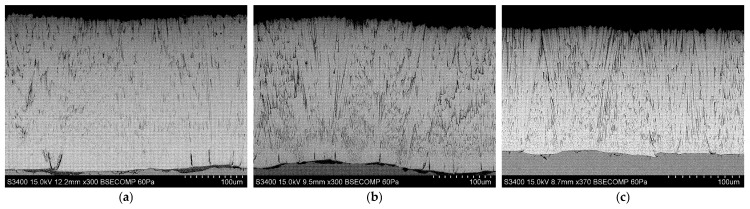
SEM cross-sectional morphology of the 7YSZ top coat deposited on the plate in location (4,2): (**a**) without plasma assistance; (**b**) with plasma assistance (100 A); (**c**) with plasma assistance (200 A).

**Figure 12:**
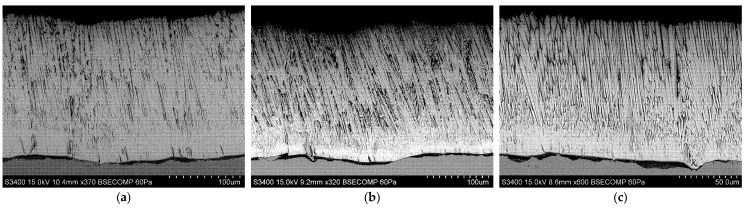
SEM cross-sectional morphology of the 7YSZ top coat deposited on the plate in location (6,2): (**a**) without plasma assistance; (**b**) with plasma assistance (100 A); (**c**) with plasma assistance (200 A).

**Figure 13:**
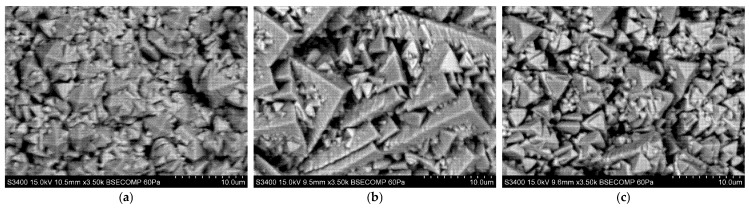
SEM topography of the 7YSZ top coat deposited on the plate in location (2,2): (**a**) without plasma assistance; (**b**) with plasma assistance (100 A); (**c**) with plasma assistance (200 A).

**Figure 14:**
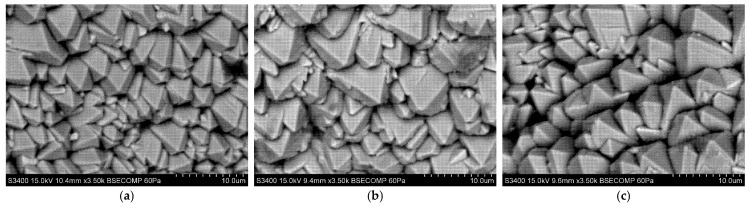
SEM topography of the 7YSZ top coat deposited on the plate in location (4,2): (**a**) without plasma assistance; (**b**) with plasma assistance (100 A); (**c**) with plasma assistance (200 A).

**Figure 15:**
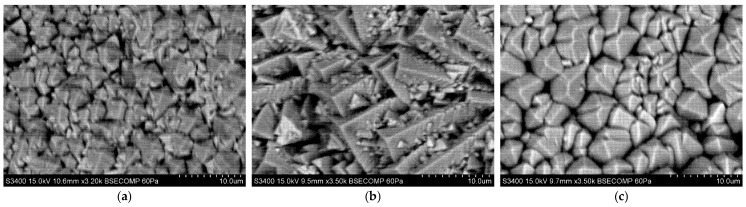
SEM topography of the 7YSZ top coat deposited on the plate in location (6,2): (**a**) without plasma assistance; (**b**) with plasma assistance (100 A); (**c**) with plasma assistance (200 A).

Figure 10, Figure 11 and Figure 12 show the columnar microstructure of the ceramic coatings revealed in cross-sectional view, while Figure 13, Figure 14 and Figure 15 display the surface topographies of the as-deposited coatings. The cross-sectional images clearly illustrate the effect of plasma current on the coating thickness. Although coatings deposited without plasma and with a plasma current of 100 A exhibit comparable thicknesses, a further increase in plasma current to 200 A results in a significant reduction in coating thickness. In addition to thickness variation, distinct changes in column morphology are evident. As the plasma current increases, the columnar structure becomes more compact. In this work, “denser” denotes a more compact column morphology with well-defined boundaries and a reduced proportion of fine, feather-like surface crystallites, as apparent in both cross-sectional and surface images. The influence of substrate temperature on coating morphology is also distinctly evident. At the outermost positions (Figure 10 and Figure 12), the coatings exhibit noticeably lower column packing density compared to those obtained at the intermediate position (Figure 11). This difference arises from the processing conditions: The coatings at the intermediate position (4,2) were deposited at 950 °C without plasma assistance and 850 °C with plasma assistance, while the coatings at the outermost positions were deposited at lower substrate temperatures, namely 900 °C without plasma assistance and 800 °C with plasma assistance. The observed effect can be attributed to the use of one single active crucible during all deposition experiments, which results in the highest vapour flux density being concentrated directly above the crucible. Consequently, the flux intensity decreases with increasing lateral distance from the evaporation source, leading to a reduced apparent column packing at the peripheral positions. All images were recorded at identical magnifications and imaging settings at matched substrate positions to ensure a like-for-like comparison.

Surface topography images further support these observations. In all analysed positions, increasing the plasma current leads to a visible reduction in the proportion of fine surface crystallites, often associated with the growth of loosely packed, feather-like columns. This trend is consistent with the microstructural changes seen in cross-sections. At the outermost positions (Figure 13 and Figure 15), a systematic variation with plasma current is observed. In the absence of plasma assistance, the surface is dominated by fine crystallites with rounded tips. With a plasma current of 100 A, the crystallites become significantly larger and display sharper, more angular tips. At 200 A, the crystallite size decreases compared to the 100 A case, yet remains larger than in the plasma-free condition, while the tips retain their sharp and well-defined shape. A similar, though less pronounced, evolution is observed at the intermediate position (Figure 14), further confirming the influence of plasma assistance on surface morphology and growth dynamics. These trends are consistent with the combined effect of plasma current and local temperature/flux conditions inherent to the single-crucible layout. Moreover, at the intermediate position (4,2), the effect of the higher substrate temperature is clearly manifested in the increased regularity and uniform orientation of the crystallites, in contrast to the less ordered structures observed at the outermost positions ((2,2) and (6,2)).

### 3.3. TBC Coating Scratch Resistance

Scratch tests were conducted to evaluate the mechanical integrity and adhesion of the coatings deposited by electron beam physical vapour deposition, both with and without hollow cathode plasma assistance. Measurements were made on specimens extracted from the intermediate position (4,2) of coated plates. Under the applied testing conditions, no delamination or coating spallation was observed for any of the specimens, confirming excellent adhesion of the top coat in both standard and plasma-assisted deposition modes and highlighting its suitability for TBC applications.

**Figure 16:**
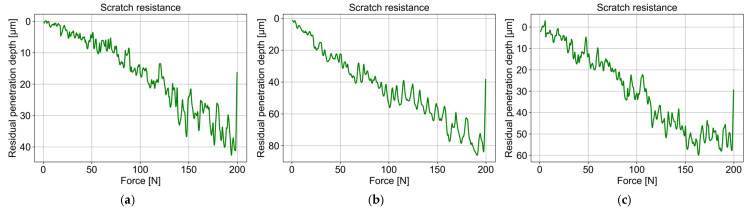
Scratch resistance of the 7YSZ top coat deposited on the plate in location (4,2): (**a**) without plasma assistance; (**b**) with plasma assistance (100 A); (**c**) with plasma assistance (200 A).

Analysis of the residual penetration depth (Figure 16) revealed that, under a load of 200 N, the diamond indenter penetrated the coating deposited without plasma assistance to a depth of approximately 40 µm. In contrast, coatings deposited with hollow cathode plasma assistance exhibited higher penetration depths: 80 µm at a plasma current of 100 A and 60 µm at 200 A. These findings indicate that plasma-assisted coatings deposited at 850 °C exhibit higher apparent brittleness relative to coatings deposited at 950 °C without plasma assistance. This suggests that within the studied process window, the additional energy supplied by the hollow cathode plasma is insufficient to fully compensate for the reduced substrate temperature, leading to microstructural modifications that downgrade the coating’s resistance to mechanical loading. This finding underscores the critical role of substrate temperature and energy input in controlling both the microstructure and mechanical performance of EB-PVD thermal barrier coatings.

## 4. Conclusions

This study demonstrated that the application of hollow cathode plasma assistance in the electron beam physical vapour deposition (EB-PVD) process does not significantly affect the spatial distribution of the ceramic coating thickness across the substrate. Under all tested conditions, the maximum thickness consistently occurred above the evaporation source, and the thickness gradually decreased towards the plate edges. This suggests that the distribution of the vapour flux is primarily influenced by the relative positioning of the crucible and substrate, although the contribution of plasma-related effects cannot be fully excluded and may become more apparent under different EB-PVD process configurations. On the contrary, plasma assistance was found to have a pronounced effect on the absolute thickness of the deposited ceramic coatings. Notably, the application of a 200 A plasma current resulted in a substantial decrease in coating thickness compared to the plasma-free process. Plasma assistance also exerted a clear influence on the microstructure and surface morphology of the coatings. Cross-sectional SEM images revealed a transition toward denser, more compact columnar structures with increasing plasma current. Simultaneously, surface observations showed a reduced presence of fine surface crystallites and the development of grains with sharper, more angular tips. These effects were most pronounced at peripheral regions of the substrate, areas subject to lower temperatures during deposition, underscoring the role of plasma in enhancing surface diffusion and altering growth dynamics under suboptimal thermal conditions. X-ray diffraction (XRD) confirmed the presence of stabilised tetragonal zirconia (tetragonal-YSZ) in all coatings, independent of plasma conditions. This phase is widely recognised for its favourable thermal and mechanical properties and is considered a desirable constituent in advanced thermal barrier coating systems (TBCs). Importantly, coatings obtained with plasma assistance at 100 A using a reduced electron beam current of 1.82 A exhibited thickness and morphology comparable to those obtained without plasma at a higher current of 2.32 A, even though these coatings were deposited at a process temperature approximately 100 °C lower. However, scratch tests indicated that plasma-assisted coatings deposited at 850 °C exhibit an increased apparent brittleness compared to coatings deposited at 950 °C without plasma assistance. This finding suggests that, in future studies, elevating the substrate temperature during the plasma-assisted EB-PVD process may be necessary to enhance the mechanical performance and reduce the brittleness of the resulting coatings.

In summary, the integration of hollow cathode plasma into the EB-PVD process represents a promising enhancement to the conventional technique. It offers the potential for improved energy efficiency, reduced process temperature, and broader applicability to thermally sensitive substrates, thereby opening new possibilities for the development and industrial implementation of next-generation thermal barrier coating systems.

## Figures and Tables

**Table 1:** EB-PVD process trials parameters.

Trial Number	Hollow-Cathode Plasma Current [A]	Emission Current [A]	Pressure [mbar]	Temperature [°C]	Feed Rate [mm/min]	Duration [s]
01	100	1.82	0.00991	841	1.5	1200
02	200	1.62	0.00969	858	1.5	1200
03	0	2.32	0.00997	947	1.5	1200

**Table 2:** Comparison of the coating thickness obtained from non-destructive measurements from the thickness gauge and cross-sectional measurements conducted using SEM.

Location of Measurement *	Thickness of the Coating [µm] Deposited Without Plasma Assistance Measured By		Thickness of the Coating [µm] Deposited with Plasma Assistance (100 A) Measured By		Thickness of the Coating [µm] Deposited with Plasma Assistance (200 A) Measured By	
	Non-Destructive Method	SEM Cross-Section Analysis	Non-Destructive Method	SEM Cross-Section Analysis	Non-Destructive Method	SEM Cross-Section Analysis
(1,1)	164	162	147	144	85	84
(1,2)	166	163	155	158	94	94
(1,3)	159	158	153	158	97	97
(2,1)	192	188	200	213	120	118
(2,2)	203	204	214	213	138	133
(2,3)	189	187	209	200	137	132
(3,1)	245	233	231	230	144	154
(3,2)	249	235	258	240	162	168
(3,3)	236	225	252	249	161	169
(4,1)	256	254	245	233	151	156
(4,2)	265	257	265	248	165	170
(4,3)	252	238	261	243	163	160
(5,1)	241	235	240	239	138	146
(5,2)	244	241	265	249	153	160
(5,3)	238	224	262	250	150	156
(6,1)	206	193	195	201	111	113
(6,2)	214	200	210	211	125	118
(6,3)	199	185	206	198	122	119
(7,1)	156	157	145	151	72	74
(7,2)	153	156	157	167	86	89
(7,3)	146	151	155	158	85	87

* Coordinates as per Figure 3.

## Data Availability

The original contributions presented in this study are included in the article. Further inquiries can be directed to the corresponding author.

## Abbreviations

The following abbreviations are used in this manuscript:

TBC	Thermal Barrier Coating
EB-PVD	Electron Beam-Physical Vapour Deposition
HC	Hollow Cathode
SEM	Scanning Electron Microscopy
